# Temporal clustering of neuroblastic tumours in children and young adults from Northern England

**DOI:** 10.1186/s12940-015-0058-z

**Published:** 2015-09-04

**Authors:** Colin R. Muirhead, Deborah A. Tweddle, Nermine O. Basta, Richard J. Q. McNally

**Affiliations:** Institute of Health & Society, Newcastle University, Baddiley-Clark Building, Richardson Road, Newcastle upon Tyne, NE2 4AX UK; Northern Institute for Cancer Research, Newcastle University, Newcastle upon Tyne, UK

**Keywords:** Epidemiology, Neuroblastic tumours, Temporal clustering

## Abstract

**Background:**

The aetiology of neuroblastic tumours is unclear with both genetic and environmental factors implicated. The possibility that an infectious agent may be involved has been suggested. ‘Temporal clustering’ occurs if cases display an irregular temporal distribution and may indicate the involvement of an agent that exhibits epidemicity. We tested for the presence and nature of temporal clustering using population-based data from northern England.

**Methods:**

We extracted all cases of neuroblastic tumours diagnosed in children and young adults aged 0–24 years during 1968–2011 from the Northern Region Young Persons’ Malignant Disease Registry. This is a population-based registry, covering a population of approximately 900,000 young persons, and includes all cases resident in northern England at the time of diagnosis. Tests for temporal clustering were applied using a modified version of the Potthoff-Whittinghill method. Estimates of extra-Poisson variation ($\widehat{\upbeta}$) and standard errors (SEs) were obtained.

**Results:**

227 cases of neuroblastic tumours were diagnosed during the study period. All the analyses between fortnights and between months found significant extra-Poisson variation, with $\widehat{\upbeta}$ =0.846 (SE = 0.310, *P* = 0.004) for the analysis between fortnights within months. Restricting the analyses to the 76 cases diagnosed at ages less than 18 months showed significant extra-Poisson variation between fortnights within months ($\widehat{\upbeta}$ =1.532, SE = 0.866, *P* = 0.038), but not between months. In contrast, analyses of cases aged 18 months to 24 years showed significant extra-Poisson variation between quarters within years, as well as over shorter timescales.

**Conclusions:**

Transient environmental agents may be involved in the aetiology of neuroblastic tumours. The initiating factor might be a geographically-widespread agent that occurs in ‘mini-epidemics’.

**Electronic supplementary material:**

The online version of this article (doi:10.1186/s12940-015-0058-z) contains supplementary material, which is available to authorized users.

## Background

Both genetic and environmental factors are likely to be involved in the aetiology of neuroblastic tumours. Increased risk has been associated with congenital abnormalities, germline *ALK* and *PHOX2B* gene mutations, gestational diabetes, both high and low birth weight, maternal anaemia during pregnancy and neonatal respiratory distress with a low 1-min Apgar score [1–10]. A number of studies have suggested various environmental risk factors. Both prenatal and early-life exposures have been postulated. In some (but not all) studies an increased risk of neuroblastic tumours has been reported for families with higher numbers of siblings, parental alcohol consumption, maternal hair dye use, vaginal infections during pregnancy, paternal exposure to certain chemicals, parental occupational electromagnetic field exposure, other parental occupational exposures, residential exposure to pesticides, ambient air toxic exposures during pregnancy, maternal use of certain medications or recreational drugs and smoking during pregnancy [11–28]. In contrast, other studies found protective effects for childhood infectious diseases, breast-feeding, prenatal vitamin supplements, maternal history of previous fetal loss, folic acid food fortification and allergic disease, and Down Syndrome in the child [12, 29–35].

The present study is focused on the detection of irregular temporal occurrences of neuroblastic tumours. A general irregular temporal distribution of cases that is not limited to one particular time of year is known as ‘temporal clustering’. This type of clustering could arise because there are a small number of periods with greatly increased incidence or a large number of periods with moderately increased incidence. The presence of such clustering would be consistent with the involvement of a temporally varying environmental agent in aetiology. Putative exposures include atmospheric pollutants and infections. It is plausible that, in certain circumstances, infections may be both a risk factor and also convey protection (e.g., by immune stimulation). Thus it would be possible that infections may contribute to both temporal excesses and deficits. We used a recently developed methodology to analyse population-based data on incident cases of neuroblastic tumours from a region that has a relatively stable population, with ethnic homogeneity and low rates of migration into or out of the area [36–38].

## Methods

All cases aged 0–24 years, diagnosed with a neuroblastic tumour (neuroblastoma, ganglioneuroblastoma or ganglioneuroma) during the period 1968–2011 were extracted from the Northern Region Young Persons’ Malignant Disease Registry (NRYPMDR). This specialist registry was initiated in 1968 and includes the geographical area comprising the counties of Northumberland, Tyne and Wear, Durham, Teesside, and Cumbria (excluding Barrow-in–Furness) in northern England. The population of the study area aged 0–24 years is approximately 900,000 [39]. All malignancies in patients aged 0–24 years, resident in the region, are reported to the registry with a number of different sources used to identify patients. Malignancies are notified to the registry by consultants throughout the region and there are regular cross-checks of data with regional and national cancer registries. The NRYPMDR has very good completeness and ascertainment (>98 %) [40].

The data collected by the registry are exempt from individual patient consent originally under Section 60 of the UK Health and Social Care Act 2001, which has now been superseded by Section 251 of the National Health Service Act 2006. Ethical review was not required for the present study, as the patient data used were deemed to be non-identifiable.

### Prior hypothesis

The following aetiological hypothesis was tested: a primary factor influencing the temporal occurrence of neuroblastic tumours is related to exposure to a geographically widespread, irregularly temporally varying environmental agent occurring either close to diagnosis or at similar times before diagnosis.

### Statistical analysis

The statistical methods were similar to those used in a previous analysis of temporal clustering of disease [41]. A modified version [42] of the Potthoff-Whittinghill technique [43, 44] was used to assess the degree of extra-Poisson variation in the numbers of cases of neuroblastic tumour per fortnight, calendar month, quarter of a year and calendar year. Specifically, the numbers of cases were assumed to follow a negative binomial distribution, with the ratio of the variance to the expected number of cases equal to a constant, namely 1 + β. If β equals 0, then the numbers of cases are distributed as Poisson, whereas if β is greater than 0, then the numbers of cases exhibit extra-Poisson variation, i.e., are over-dispersed relative to Poisson. The estimate of extra-Poisson variation, namely $\widehat{\upbeta}$, and its standard error were based on the score statistic [42]. One-sided tests were conducted for the presence of extra-Poisson variation (i.e., β > 0), with *P* values assessed using 10000 simulations assuming β equal to zero. Findings with *P* < 0.05 were judged to be statistical significant, while values of *P* between 0.05 and 0.10 were judged to provide weak evidence of an effect.

In order to remove the effects of longer-term variation when looking for any short-term effects, analyses were conducted conditional on the total number of cases within a calendar month, quarter of a year (defined as January to March; April to June; July to September; and October to December), calendar year or total study period. Particular attention was directed towards analyses based on the following hierarchy: between fortnights within calendar months; between months within quarters of a year; between quarters within years; and between years. Since the analysis between fortnights within months was conducted conditional on the total number of cases in each month, the results from this analysis are independent of those from the analysis between months within quarters; the same applies for the other analyses in this hierarchy, meaning that these analyses can be interpreted independently of each other. Descriptive analyses showed a preponderance of cases with a diagnosis date of the 15^th^ of the month, reflecting instances where the day of the month was unknown. Consequently, fortnights were defined pragmatically as the first 14 days of the calendar month versus the remainder of the month from the 16^th^ onwards, i.e., excluding the twelve cases with a diagnosis date of the 15^th^. However, since there is no reason to think that the month or year of diagnosis is incorrect, all of the cases were included in the analyses between months, between quarters and between years.

The expected number of cases was assumed to be proportional to the length of the period in question. These expected numbers were standardised on the basis that their total would equal the total number of cases observed during the relevant period. For analyses of extra-Poisson variation between years, it was not possible to take account of year-on-year variations in population size because of a lack of data for the relevant age range within the study area over the full study period. However, based on population data for much of the study area over 1987–2003 and estimated population sizes for other years, sensitivity analyses were performed in which expected numbers of cases per year were assumed to be proportional to these population sizes. No such adjustments were necessary for analyses within years, since any variation in population size within years is likely to be minimal.

Sensitivity analyses were also conducted for cases occurring at ages less than 18 months, given that they form a notable proportion of the cases diagnosed at ages under 25 years; for cases diagnosed at ages between 18 months and 24 years inclusive; for males and females separately; and including diagnoses of other peripheral nervous system tumours [see Additional file 1].

## Results

**Fig. 1:**
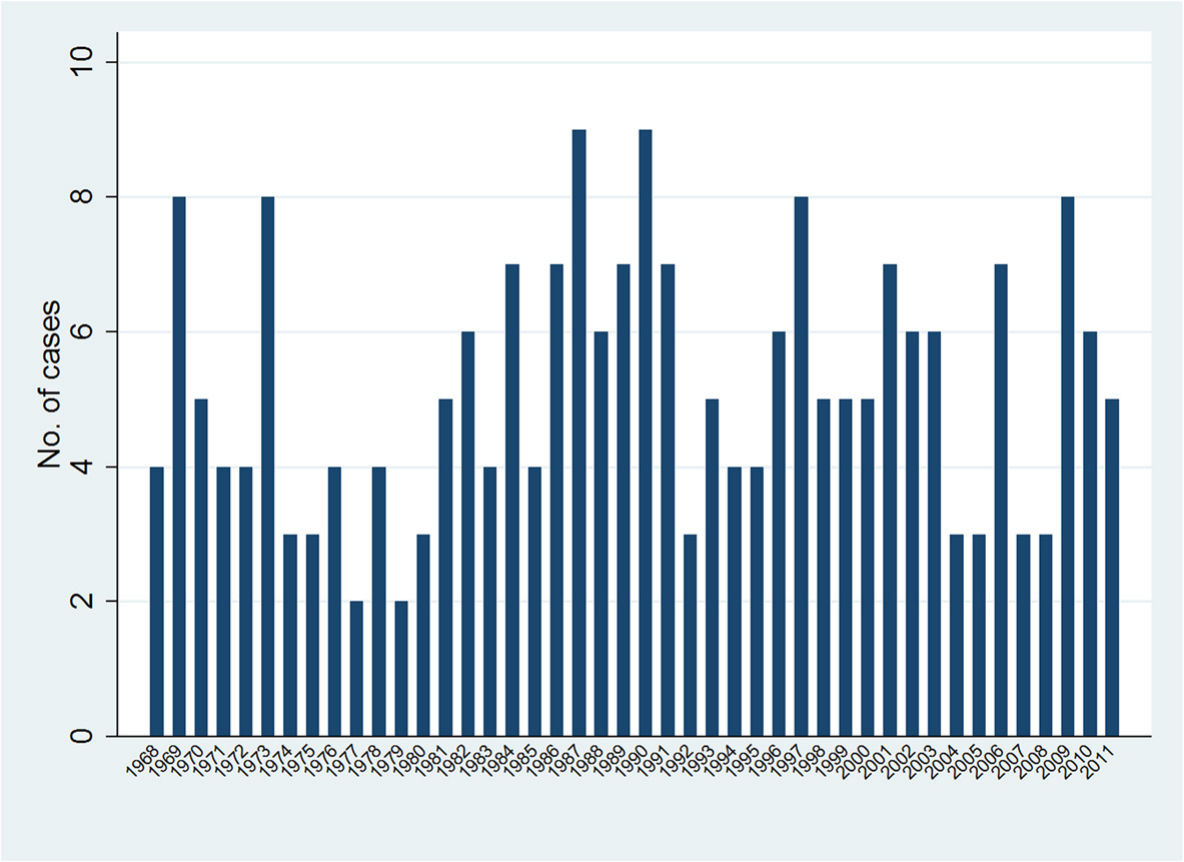
Numbers of cases per year of neuroblastic tumours at ages 0–24 years in the NRYPMDR. Shown are the numbers of cases by calendar year of diagnosis

A total of 227 cases aged 0–24 years were diagnosed in the study area during 1968–2011. Table 1 provides a breakdown of these cases by gender, age at diagnosis and period of diagnosis. Figure 1 shows the number of cases by year of diagnosis. No long-term pattern is evident, as confirmed by the Potthoff-Whittinghill analyses between years and between quarters of years (see lower part of Table 2); sensitivity analyses based on estimated annual population sizes gave similar results. In contrast, all of the analyses between fortnights and between months found significant extra-Poisson variation, with $\widehat{\upbeta}$ equal to 0.495 (SE 0.165, *P* = 0.002) for the analysis between months within quarters and 0.846 (SE 0.310, *P* = 0.004) for the analysis between fortnights within months. Restricting the analyses to the 76 cases diagnosed at ages less than 18 months also did not show significant evidence of extra-Poisson variation between months, between quarters or between years. However, there was significant extra-Poisson variation between fortnights (see Table 3), with $\widehat{\upbeta}$ equal to 1.532 (SE = 0.866, *P* = 0.038) for the analysis between fortnights within months. For the 151 cases diagnosed at ages 18 months or later, there was significant extra-Poisson variation between quarters within years ($\widehat{\upbeta}$ = 0.304, SE = 0.161, *P* = 0.035), as well as between fortnights and between months (see Table 4).

**Table 1:** Characteristics of cases of neuroblastic tumour in the NRYPMDR^a^

		No. of cases (%)	
Overall (0–24 years)		227 (100 %)	
Males		118 (52 %)	
Females		109 (48 %)	
Age at diagnosis:			
<18 months		76 (33.5 %)	
18 months – 4 years		99 (43.6 %)	
5–24 years		52 (22.9 %)	
Time period:			
1968–1978		49 (21.6 %)	
1979–1989		60 (26.4 %)	
1990–2000		61 (26.9 %)	
2001–2011		57 (25.1 %)	
	Mean	Median	Inter-quartile range
Age at diagnosis (years)	3.74	2.23	0.96–4.51
Annual number of cases	5.16	5	4–7

^a^Based on cases diagnosed during 1968-2011 inclusive

**Table 2:** Analyses of temporal clustering of neuroblastic tumours at ages 0–24 years in the NRYPMDR^a^

	$\widehat{\upbeta}$ ^b^ (SE)^c^			
	*one-sided P-value* ^*d*^			
Type of analysis	Within months	Within quarters	Within years	Within full study period
Between fortnights^e^	0.846 (0.310)	0.501 (0.108)	0.201 (0.051)	0.168 (0.044)
	*P = 0.004*	*P < 0.001*	*P < 0.001*	*P < 0.001*
Between months		0.495 (0.165)	0.221 (0.073)	0.154 (0.062)
		*P = 0.002*	*P = 0.002*	*P = 0.008*
Between quarters			0.201 (0.140)	0.020 (0.107)
			*P = 0.087*	*P = 0.39*
Between years				−0.299 (0.216)
				*P = 0.92*

a)Based on cases diagnosed during 1968–2011 inclusive

b)$\widehat{\upbeta}$ is the one-step estimate of β, the extra-Poisson variation, calculated as S/i(0) in the notation of Muirhead [42]

c)SE is the standard error of $\widehat{\upbeta}$ in the absence of extra-Poisson variation, calculated as 1/√i(0) in the notation of Muirhead [42]

d)*P*-values have been calculated using 10000 simulations, assuming Poisson variation. All *P*-values are one-sided

e)Cases with a diagnosis date of the 15^th^ of the month have been excluded from the analyses between fortnights but have been included in the other analyses

**Table 3:** Analyses of temporal clustering of neuroblastic tumours at ages <18 months in the NRYPMDR^a^

	$\widehat{\upbeta}$ ^b^ (SE)^c^			
	*one-sided P-value* ^*d*^			
Type of analysis	Within months	Within quarters	Within years	Within full study period
Between fortnights^e^	1.532 (0.866)	0.562 (0.255)	0.215 (0.082)	0.097 (0.044)
	*P = 0.038*	*P = 0.018*	*P = 0.016*	*P = 0.025*
Between months		0.300 (0.387)	0.133 (0.118)	0.043 (0.062)
		*P = 0.18*	*P = 0.12*	*P = 0.18*
Between quarters			0.214 (0.226)	−0.032 (0.108)
			*P = 0.17*	*P = 0.54*
Between years				−0.403 (0.217)
				*P = 0.98*

a)Based on cases diagnosed during 1968–2011 inclusive

b)$\widehat{\upbeta}$ is the one-step estimate of β, the extra-Poisson variation, calculated as S/i(0) in the notation of Muirhead [42]

c)SE is the standard error of $\widehat{\upbeta}$ in the absence of extra-Poisson variation, calculated as 1/√i(0) in the notation of Muirhead [42]

d)*P*-values have been calculated using 10000 simulations, assuming Poisson variation. All *P*-values are one-sided

e)Cases with a diagnosis date of the 15^th^ of the month have been excluded from the analyses between fortnights but have been included in the other analyses

**Table 4:** Analyses of temporal clustering of neuroblastic tumours at ages 18 months to 24 years inclusive in the NRYPMDR^a^

	$\widehat{\upbeta}$ ^b^ (SE)^c^			
	*one-sided P-value* ^*d*^			
Type of analysis	Within months	Within quarters	Within years	Within full study period
Between fortnights^e^	0.754 (0.417)	0.434 (0.145)	0.163 (0.060)	0.123 (0.044)
	*P = 0.036*	*P = 0.003*	*P = 0.008*	*P = 0.008*
Between months		0.367 (0.215)	0.172 (0.084)	0.128 (0.062)
		*P = 0.046*	*P = 0.027*	*P = 0.023*
Between quarters			0.304 (0.161)	0.143 (0.107)
			*P = 0.035*	*P = 0.091*
Between years				−0.060 (0.216)
				*P = 0.56*

a)Based on cases diagnosed during 1968–2011 inclusive

b)$\widehat{\upbeta}$ is the one-step estimate of β, the extra-Poisson variation, calculated as S/i(0) in the notation of Muirhead [42]

c)SE is the standard error of $\widehat{\upbeta}$ in the absence of extra-Poisson variation, calculated as 1/√i(0) in the notation of Muirhead [42]

d)*P*-values have been calculated using 10000 simulations, assuming Poisson variation. All *P*-values are one-sided

e)Cases with a diagnosis date of the 15^th^ of the month have been excluded from the analyses between fortnights but have been included in the other analyses

Analyses by gender (Table 5) showed that the evidence for extra-Poisson variation at ages 0–24 years was largely restricted to the 118 cases among males. In particular, $\widehat{\upbeta}$ was significantly greater among males than among females in all of the analyses between fortnights and between months, apart from the analysis between fortnights within months where the evidence for extra-Poisson variation was stronger among females (*P* = 0.004) than among males (*P* = 0.12). Furthermore, there was evidence of extra-Poisson variation in male diagnoses between quarters within years (*P* = 0.003), but not among females (*P* = 0.39). Further analysis showed that the evidence of extra-Poisson variation among cases diagnosed at ages less than 18 months was restricted to males [see Additional file 2], whereas there tended to be less evidence of differences in $\widehat{\upbeta}$ between males and females based on cases diagnosed at age 18 months or older [see Additional file 3].

**Table 5:** Analyses of temporal clustering of neuroblastic tumours at ages 0-24 years, separately for males and females in the NRYPMDR^a^

		$\widehat{\upbeta}$ ^b^ (SE)^c^			
		*one-sided P-value* ^*d*^			
Type of analysis		Within months	Within quarters	Within years	Within full study period
Between fortnights^e^	Males	0.507 (0.423)	0.541 (0.162)	0.297 (0.063)	0.150 (0.044)
		P = 0.12	P = 0.002	P < 0.001	P = 0.002
	*Females*	*2.095 (0.894)*	*0.157 (0.194)*	*0.036 (0.068)*	*0.005 (0.044)*
		*P = 0.004*	*P = 0.19*	*P = 0.29*	*P = 0.39*
Between months	Males		0.831 (0.248)	0.417 (0.089)	0.204 (0.062)
			P < 0.001	P < 0.001	P = 0.002
	*Females*		*−0.160 (0.282)*	*−0.027 (0.095)*	*−0.058 (0.062)*
			*P = 0.69*	*P = 0.59*	*P = 0.78*
Between quarters	Males			0.508 (0.170)	0.134 (0.107)
				P = 0.003	P = 0.10
	*Females*			*0.039 (0.181)*	*−0.064 (0.107)*
				*P = 0.39*	*P = 0.68*
Between years	Males				−0.435 (0.217)
					P = 0.99
	*Females*				*−0.185 (0.217)*
					*P = 0.79*

a)Based on cases diagnosed during 1968–2011 inclusive

b)$\widehat{\upbeta}$ is the one-step estimate of β, the extra-Poisson variation, calculated as S/i(0) in the notation of Muirhead [42]

c)SE is the standard error of $\widehat{\upbeta}$ in the absence of extra-Poisson variation, calculated as 1/√i(0) in the notation of Muirhead [42]

d)*P*-values have been calculated using 10000 simulations, assuming Poisson variation. All *P*-values are one-sided

e)Cases with a diagnosis date of the 15^th^ of the month have been excluded from the analyses between fortnights but have been included in the other analyses

Including the 22 cases of other peripheral nervous cell tumours (as defined in Additional file 1) diagnosed in the study area at ages 0–24 years together with the 227 cases of neuroblastic tumours tended to weaken the evidence for extra-Poisson variation in the analyses between fortnights and between months, with lower values for $\widehat{\upbeta}$ than before, e.g., 0.403 (SE = 0.153, *P* = 0.005) for the analysis between months within quarters and 0.645 (SE = 0.289, *P* = 0.015) for the analysis between fortnights within months. However, as before, all of the analyses between fortnights and between months found significant extra-Poisson variation (results not shown).

## Discussion

This analysis has found evidence of temporal variation of neuroblastic tumours in children and young adults over short periods. After allowing for annual differences, the numbers of cases in each quarter were as expected under the Poisson assumption (i.e., no evidence of extra-Poisson variation), although there was evidence of extra-Poisson variation between quarters within years among males and at ages 18 months or older. Conversely the number of cases in each fortnight and month showed greater variation than would be expected under the Poisson distribution (i.e., evidence of extra-Poisson variation). These findings suggest that temporal clustering of cases occurs in short periods of one month or less, so indicating the involvement of transient environmental agents in aetiology. Furthermore, the magnitude of the extra-Poisson variation was lower when cases of other peripheral nervous cell tumours were added to the analysis, suggesting that the temporal clustering reported here is specific to neuroblastic tumours rather than to peripheral nervous cell tumours as a whole.

To our knowledge there have been no previous studies of temporal clustering of neuroblastic tumours. However, both spatial clustering and space-time clustering have been analysed in earlier studies. Spatial clustering is defined as an irregular spatial distribution of cases of a disease and is interpreted as suggesting an environmental aetiology. Space-time clustering is defined as an irregular distribution of cases of a disease in time and space and is interpreted as suggesting an environmental, possibly infectious aetiology. Previous studies from the UK have not found evidence of space-time (based on both birth and diagnosis) or spatial clustering (based on diagnosis) amongst cases of childhood neuroblastic tumours [45–48]. An earlier study from the USA found evidence of space-time clustering, based on birth but not diagnosis [49], and a more recent study from Spain found evidence of space-time clusters, based on diagnosis [50]. The lack of consistency in finding space-time clustering for neuroblastic tumours may indicate that an environmental agent only precipitates the onset of these tumours in rare circumstances, with cases diagnosed at disparate locations. However, the finding of temporal clustering suggests that – within periods up to a few months in length - diagnoses tend to occur at similar times. It should be stressed that there is no reason to believe that these results are an artefact linked to access to health care; specifically, the organisation of primary care in England means that possible cases would present and be referred to specialists without any tendency to cluster over time.

A notable aspect of our findings was that – with the exception of the analyses between fortnights within months - the evidence for temporal clustering was largely restricted to males. In particular, for the analyses between months, the estimate of extra-Poisson variation was significantly greater among males than among females. We had no prior expectation that the level of temporal clustering would differ by gender. Furthermore, previous studies have not suggested that the aetiology of childhood neuroblastic tumours differs between males and females, although they have indicated that such tumours are about 20 % more common among males than among females [51, 52]. Consequently, our findings on possible differences in temporal clustering by gender should be interpreted with caution.

About a third of the cases studied here were diagnosed in the first 18 months of life. Within this age group, there was no evidence of temporal clustering between periods of a month or more. However, temporal clustering was seen between fortnights within months. This suggests that a transient agent may be involved in aetiology and – given the narrow age range – that exposure to this agent arose shortly before diagnosis. In particular, exposure to such an agent might act as a final “trigger” in the carcinogenic process and lead to a clinically-recognisable tumour very soon thereafter. In contrast, analysis of cases diagnosed at ages between 18 months and 24 years found greater evidence of temporal clustering over longer periods; specifically, between quarters within years. An earlier study in Germany [53] indicated possible differences in the aetiology of childhood neuroblastic tumours between early and late-stage disease at time of diagnosis. This raises the possibility that temporal clustering may be more of a feature of high-risk rather than low-risk disease. This topic will be addressed further in a future study. The suggestion of differences between the results for ages less than 18 months and those for older ages might indicate that the lag time between any relevant exposure and diagnosis of the disease varies by age. That said, the majority of cases diagnosed after the first 18 months of life arose before 5 years (see Table 1), so indicating that even for these cases the lag period is likely to be relatively short; specifically, of the order of months rather than years. This would fit in with the findings for this older age group.

It is important to note that the present study did not analyse seasonal variation, i.e., whether neuroblastic tumours diagnoses tend to occur more often than expected in the same month of the same season each year. Instead, we focused on whether diagnoses tend to cluster together in time, without regard for any periodicity in the pattern of occurrence. A few previous studies have considered analyses of seasonality. A major study from the USA did not find seasonal variation in month of diagnosis [54]. Another study from Italy did not find overall seasonal variation of date of diagnosis or birth, but this study did report seasonal variation for birth in infants with stage 4 s disease [55]. Within the UK, a study based on the NRYPMDR did not find any evidence of seasonality by either month of birth or month of diagnosis in cases of sympathetic nervous system tumours, most of which are neuroblastic tumours [56].

The present study has several limitations. By its nature, it is not possible to identify specific agents that might be involved in the aetiology of neuroblastic tumours, although it does indicate facets of such agents (see below). Information was not available on annual population sizes for the full study period. However, sensitivity analyses based on estimated population sizes gave similar results to those reported here; also, the analyses within years are unaffected by variations in annual population sizes. It might also be queried whether the quality of the data has varied over the 44 year period of this study. However, a relatively recent histological review conducted by one of the authors (DT) confirmed all of the cases as being neuroblastic tumours. Furthermore, the diagnoses of neuroblastic tumours have been made in accordance with internationally agreed criteria [57]. In any case, any long-term changes are unlikely to influence analyses within periods of a year or less.

Since neuroblastic tumours are rare, the reliability of the findings might be queried on the basis of the low annual number of cases (about 5 per year in the study area) and the possibility of errors in some dates of diagnosis. However, this is not supported by differences between the findings at ages less than 18 months and those at older ages, which indicate longer lag times at older ages. Furthermore, analyses carried out of other cancers in the NRYPMDR using the same methodology have – in most instances – not found temporal clustering of the type reported here, despite often being based on similar numbers of cases (unpublished results). Taken together with the common approach used to define diagnosis dates in this dataset, it is unlikely that our results are due to data errors.

Our study has found that neuroblastic tumours occur in ‘mini-epidemics’ that are geographically widespread and occur at specific points in time. These findings support our prior hypothesis that a primary factor influencing the temporal incidence of neuroblastic tumours is related to exposure to an irregularly temporally varying environmental agent occurring close to diagnosis or at similar times before diagnosis. Examples of widespread transient agents include infections and atmospheric pollution. It is interesting to note that one study has suggested that infections confer a protective effect on the development of neuroblastic tumours [29]. Such an effect could lead to temporal deficits in incidence and thus the occurrence of temporal clustering. This is important in view of evidence that immunological surveillance might be associated with the regression of stage 4 s neuroblastic tumours [58, 59] and that advanced, aggressive cases of neuroblastic tumours evade immunological surveillance [60, 61]. In contrast, a recent study has found that exposure to ambient air toxics (carbon tetrachloride, polycyclic aromatic hydrocarbons and hexavalent chromium) during pregnancy was associated with an increased risk of neuroblastic tumours [23], although there is currently little other evidence to support a role for atmospheric pollution.

In conclusion, this study found new evidence for general irregular (non-seasonal) temporal clustering among cases of neuroblastic tumours. It suggests that there is support for the involvement of an agent that occurs in geographically widespread mini-epidemics. Examples include certain common infections (e.g., influenza) and atmospheric pollution. This result now needs to be confirmed by larger national and international studies. As with the “hygiene hypothesis” for childhood leukaemia [62], future research should seek to identify specific candidate agents which could be protective or causative.

## Additional files

Additional file 1:
**The** **sub-types** **that constitute “other peripheral nervous cell tumours” and their morphological code, according to the 3** ^**rd**^ **edition of the International Classification of Childhood Cancer,** **ICCC-3.** (DOCX 19 kb) Additional file 2:
**Analyses of temporal clustering of neuroblastic tumours at ages <18 months, separately for males and females in the NRYPMDR.** (DOCX 16 kb) Additional file 3:
**Analyses of temporal clustering of neuroblastic tumours at ages 18 months to 24 years inclusive, separately for males and females in the NRYPMDR.** (DOCX 17 kb)

## Abbreviations

NRYPMDR: Northern Region Young Persons’ Malignant Disease Registry
SE: Standard Error
