# Supplementary material for: Temporal clustering of neuroblastic tumours in children and young adults from Northern England
Source: Environ Health. 2015 Sep 4;14:72. doi: 10.1186/s12940-015-0058-z (PMC4558831; doi:10.1186/s12940-015-0058-z)
Supplement: Additional file 1: — The sub-types that constitute “other peripheral nervous cell tumours” and their morphological code, according to the 3 rd edition of the International Classification of Childhood Cancer, ICCC-3. (DOCX 19 kb) [file 12940_2015_58_MOESM1_ESM.docx]

**APPENDIX 1**

The sub-types that constitute “other peripheral nervous cell tumours” and their morphological code, according to the 3^rd^ edition of the International Classification of Childhood Cancer, ICCC-3 [[1](#_ENREF_1)].

| Sub-type | Description |
| --- | --- |
| 8680/0 | Paraganglioma, benign |
| 8680/1 | Paraganglioma, NOS |
| 8680/3 | Paraganglioma, malignant |
| 8681/1 | Sympathetic paraganglioma |
| 8682/1 | Parasympathetic paraganglioma |
| 8683/0 | Gangliocytic paraganglioma (C17.0) |
| 8690/1 | Glomus jugulare tumour, NOS (C75.5)  *Jugular paraganglioma (C75.5)*  *Jugulotympanic paraganglioma (C75.5)* |
| 8691/1 | Aortic body tumour (C75.5) |
|  | *Aortic body paraganglioma (C75.5)* |
|  | *Aorticopulmonary paraganglioma (C75.5)* |
| 8692/1 | Carotid body tumour (C75.4) |
|  | *Carotid body paraganglioma (C75.4)* |
| 8693/1 | Extra-adrenal paraganglioma, NOS |
|  | Nonchromaffin paraganglioma, NOS |
|  | Chemodectoma |
| 8693/3 | Extra-adrenal paraganglioma, malignant |
|  | Nonchromaffin paraganglioma, malignant |
| 8700/0 | Pheochromocytoma, NOS (C74.1) |
|  | *Adrenal medullary paraganglioma (C74.1)* |
|  | *Chromaffin paraganglioma* |
|  | *Chromaffin tumour* |
|  | *Chromaffinoma* |
| 8700/3 | Pheochromocytoma, malignant (C74.1) |
|  | *Adrenal medullary paraganglioma, malignant (C74.1)* |
|  | *Pheochromoblastoma (C74.1)* |
| 9501/0 | Medulloepithelioma, benign (C69.4) |
|  | *Diktyoma, benign (C69._)* |
| 9501/3 | Medulloepithelioma, NOS |
|  | *Diktyoma, malignant (C69._)* |
| 9502/0 | Teratoid medulloepithelioma, benign (C69.4) |
| 9502/3 | Teratoid medulloepithelioma |
| 9503/3 | Neuroepithelioma, NOS |
| 9504/3 | Spongioneuroblastoma |

**REFERENCE**

1. Steliarova-Foucher E, Stiller C, Lacour B, Kaatsch P: **International Classification of Childhood Cancer, third edition**. *Cancer* 2005, **103**:1457-1467.
