# Supplementary material for: Temporal clustering of neuroblastic tumours in children and young adults from Northern England
Source: Environ Health. 2015 Sep 4;14:72. doi: 10.1186/s12940-015-0058-z (PMC4558831; doi:10.1186/s12940-015-0058-z)
Supplement: Additional file 2: — Analyses of temporal clustering of neuroblastic tumours at ages <18 months, separately for males and females in the NRYPMDR. (DOCX 16 kb) [file 12940_2015_58_MOESM2_ESM.docx]

**APPENDIX 2**. Analyses of temporal clustering of neuroblastic tumours at ages <18 months, separately for males and females in the NRYPMDR^a^

|  |  | $\hat{\beta}$ ^b^ (SE)^c^ | | | |
| --- | --- | --- | --- | --- | --- |
|  |  | *one-sided P-value ^d^* | | | |
| Type of analysis |  | Within months | Within quarters | Within years | Within full study period |
| Between fortnights ^e^ | Males | 0.717 (1.154) | 1.230 (0.516) | 0.388 (0.164) | 0.082 (0.044) |
|  |  | *p=0.20* | *p=0.022* | *p=0.032* | *p=0.047* |
|  | *Females* | *- ^f^* | *-0.400 (0.447)* | *-0.109 (0.130)* | *-0.036 (0.044)* |
|  |  |  | *p=0.51* | *p=0.52* | *p=0.49* |
|  |  |  |  |  |  |
| Between months | Males |  | 2.000 (0.816) | 0.363 (0.220) | 0.097 (0.062) |
|  |  |  | *p<0.001* | *p=0.050* | *p=0.042* |
|  | *Females* |  | *-1.000 (0.707)* | *-0.229 (0.188)* | *-0.074 (0.062)* |
|  |  |  | *p=0.80* | *p=0.79* | *p=0.76* |
|  |  |  |  |  |  |
| Between quarters | Males |  |  | -0.089 (0.422) | -0.044 (0.109) |
|  |  |  |  | *p=0.51* | *p=0.51* |
|  | *Females* |  |  | *0.022 (0.359)* | *-0.011 (0.108)* |
|  |  |  |  | *p=0.43* | *p=0.40* |
|  |  |  |  |  |  |
| Between years | Males |  |  |  | -0.178 (0.219) |
|  |  |  |  |  | *p=0.74* |
|  | *Females* |  |  |  | *0.009 (0.218)* |
|  |  |  |  |  | *p=0.40* |

a) Based on cases diagnosed during 1968-2011 inclusive.

b) $\hat{\beta}$ is the one-step estimate of β, the extra-Poisson variation, calculated as S/i(0) in the notation of Muirhead [42].

c) SE is the standard error of $\hat{\beta}$ in the absence of extra-Poisson variation, calculated as 1/√i(0) in the notation of Muirhead [42].

d) *P*-values have been calculated using 10000 simulations, assuming Poisson variation. All *P*-values are one-sided.

e) Cases with a diagnosis date of the 15^th^ of the month have been excluded from the analyses between fortnights but have been included in the other analyses.

f) Not calculable, since there were no months with more than one case among females.
