# Supplementary material for: Temporal clustering of neuroblastic tumours in children and young adults from Northern England
Source: Environ Health. 2015 Sep 4;14:72. doi: 10.1186/s12940-015-0058-z (PMC4558831; doi:10.1186/s12940-015-0058-z)
Supplement: Additional file 3: — Analyses of temporal clustering of neuroblastic tumours at ages 18 months to 24 years inclusive, separately for males and females in the NRYPMDR. (DOCX 17 kb) [file 12940_2015_58_MOESM3_ESM.docx]

**APPENDIX 3**. Analyses of temporal clustering of neuroblastic tumours at ages 18 months to 24 years inclusive, separately for males and females in the NRYPMDR^a^

|  |  | $\hat{\beta}$ ^b^ (SE)^c^ | | | |
| --- | --- | --- | --- | --- | --- |
|  |  | *one-sided P-value ^d^* | | | |
| Type of analysis |  | Within months | Within quarters | Within years | Within full study period |
| Between fortnights ^e^ | Males | 0.344 (0.632) | 0.274 (0.218) | 0.190 (0.077) | 0.079 (0.044) |
|  |  | *p=0.34* | *p=0.11* | *p=0.018* | *p=0.059* |
|  | *Females* | *2.152 (1.000)* | *0.416 (0.258)* | *0.151 (0.091)* | *0.072 (0.044)* |
|  |  | *p=0.002* | *p=0.057* | *p=0.068* | *p=0.055* |
|  |  |  |  |  |  |
| Between months | Males |  | 0.336 (0.335) | 0.258 (0.109) | 0.097 (0.062) |
|  |  |  | *p=0.14* | *p=0.018* | *p=0.051* |
|  | *Females* |  | *0.000 (0.374)* | *0.039 (0.125)* | *0.012 (0.062)* |
|  |  |  | *p=0.43* | *p=0.34* | *p=0.31* |
|  |  |  |  |  |  |
| Between quarters | Males |  |  | 0.526 (0.208) | 0.141 (0.108) |
|  |  |  |  | *p=0.010* | *p=0.083* |
|  | *Females* |  |  | *0.248 (0.240)* | *0.066 (0.108)* |
|  |  |  |  | *p=0.16* | *p=0.22* |
|  |  |  |  |  |  |
| Between years | Males |  |  |  | -0.298 (0.217) |
|  |  |  |  |  | *p=0.92* |
|  | *Females* |  |  |  | *0.033 (0.217)* |
|  |  |  |  |  | *p=0.39* |

a) Based on cases diagnosed during 1968-2011 inclusive.

b) $\hat{\beta}$ is the one-step estimate of β, the extra-Poisson variation, calculated as S/i(0) in the notation of Muirhead [42].

c) SE is the standard error of $\hat{\beta}$ in the absence of extra-Poisson variation, calculated as 1/√i(0) in the notation of Muirhead [42].

d) *P*-values have been calculated using 10000 simulations, assuming Poisson variation. All *P*-values are one-sided.

e) Cases with a diagnosis date of the 15^th^ of the month have been excluded from the analyses between fortnights but have been included in the other analyses.
